# Comprehensive analysis of phospholipids and glycolipids in the opportunistic pathogen *Enterococcus faecalis*

**DOI:** 10.1371/journal.pone.0175886

**Published:** 2017-04-19

**Authors:** Rafi Rashid, Amaury Cazenave-Gassiot, Iris H. Gao, Zeus J. Nair, Jaspal K. Kumar, Liang Gao, Kimberly A. Kline, Markus R. Wenk

**Affiliations:** 1 Singapore Centre on Environmental Life Sciences Engineering, Nanyang Technological University, Singapore, Singapore; 2 School of Biological Sciences, Nanyang Technological University, Singapore, Singapore; 3 Singapore Lipidomics Incubator, National University of Singapore, Singapore, Singapore; 4 Department of Biochemistry, Yong Loo Lin School of Medicine, National University of Singapore, Singapore, Singapore; 5 Life Sciences Institute, National University of Singapore, Singapore, Singapore; 6 Department of Biological Sciences, Faculty of Science, National University of Singapore, Singapore, Singapore; University of Kansas, UNITED STATES

## Abstract

*Enterococcus faecalis* is a Gram-positive, opportunistic, pathogenic bacterium that causes a significant number of antibiotic-resistant infections in hospitalized patients. The development of antibiotic resistance in hospital-associated pathogens is a formidable public health threat. In *E*. *faecalis* and other Gram-positive pathogens, correlations exist between lipid composition and antibiotic resistance. Resistance to the last-resort antibiotic daptomycin is accompanied by a decrease in phosphatidylglycerol (PG) levels, whereas multiple peptide resistance factor (MprF) converts anionic PG into cationic lysyl-PG via a trans-esterification reaction, providing resistance to cationic antimicrobial peptides. Unlike previous studies that relied on thin layer chromatography and spectrophotometry, we have performed liquid chromatography-tandem mass spectrometry (LC-MS/MS) directly on lipids extracted from *E*. *faecalis*, and quantified the phospholipids through multiple reaction monitoring (MRM). In the daptomycin-sensitive *E*. *faecalis* strain OG1RF, we have identified 17 PGs, 8 lysyl-PGs (LPGs), 23 cardiolipins (CL), 3 glycerophospho-diglucosyl-diacylglycerols (GPDGDAG), 5 diglucosyl-diacylglycerols (DGDAG), 3 diacylglycerols (DAGs), and 4 triacylglycerols (TAGs). We have quantified PG and shown that PG levels vary during growth of *E*. *faecalis in vitro*. We also show that two daptomycin-resistant (DapR) strains of *E*. *faecalis* have substantially lower levels of PG and LPG levels. Since LPG levels in these strains are lower, daptomycin resistance is likely due to the reduction in PG. This lipidome map is the first comprehensive analysis of membrane phospholipids and glycolipids in the important human pathogen *E*. *faecalis*, for which antimicrobial resistance and altered lipid homeostasis have been intimately linked.

## Introduction

The normal habitat of enterococci is the gastrointestinal tract of animals and humans. As opportunistic pathogens, enterococci can cause bacteremia, meningitis, and endocarditis, and can also infect wound sites and the urinary tract. Commonly used medical devices such as intravascular and urinary catheters provide a surface on which enterococci can easily form biofilms, which are defined as “multilayer assemblages of bacteria that are held together by a sticky polysaccharide matrix” [1]. In the clinical setting, biofilms have been implicated in an estimated 65% of nosocomial infections, and it is when bacteria are in the biofilm form that they are more resistant to conventional antibiotics [2].

*Enterococcus faecalis* is a prominent causative agent of nosocomial and opportunistic infections, and has acquired resistance to many antibiotics. Daptomycin (DAP) is an antibiotic of last resort for multidrug resistant *E*. *faecalis* [3, 4]. It is a cationic lipopeptide that requires Ca^2+^ ions to target the cell membrane. Resistance to DAP is emerging in *E*. *faecalis* and has been linked to changes in phospholipid metabolism. Genes coding for glycerophosphoryl diester phosphodiesterase *(gdpD)* and cardiolipin synthase *(cls)* have been implicated in daptomycin resistance [3, 5, 6]. Besides playing a role in antibiotic resistance, lipid metabolism is involved in biomembrane synthesis and energy homeostasis during pathogen replication and persistence [7]. Homeostasis of membrane lipids is essential to bacterial viability [8–10]. The organization of lipids and proteins into membrane microdomains also helps to coordinate various bacterial processes [11–15], which include growth, genome replication, and cell division. These processes are dependent on the subcellular organization of biological molecules, including lipids [16, 17]. Both bacterial lipid composition and lipid organization into domains are important for normal physiology, secretion, signaling, virulence, and antibiotic resistance [10–12, 18–23].

Three major classes of phospholipids occur in bacterial membranes: phosphatidylglycerol (PG), cardiolipin (CL), and phosphatidylethanolamine (PE) [24]. All three classes are derived from the common precursor phosphatidic acid (PA) [8, 25]. The predominant phospholipid species in Gram-positive bacteria are phosphatidylglycerol (PG) and cardiolipin (CL) [26], whose relative composition varies from species to species [10]. The levels of individual phospholipids were shown to depend on growth conditions [26], indicating that phospholipid regulation is very dynamic and responsive to the environment. Bacteria are able to adjust the concentration of lipids [27] and thus alter their membrane composition as an adaptation to environmental stress [8]. Depending on the immediate physiological needs of the bacterium, fatty acids may be saturated or unsaturated [28] and in *E*. *faecalis*, fatty acids in the membrane are involved in adaptation to stressful conditions. Longer fatty acid chains decrease membrane fluidity [9], while the presence of one or more double bonds in these chains will increase fluidity [8]. A decrease in temperature leads to a higher level of unsaturated fatty acids in *Bacillus subtilis* [29], which is a specific example of how lipid composition is adjusted in response to temperature [8]. Unsaturated fatty acids in phospholipids increase resistance to bile salts and the antibiotic daptomycin. For example, the unsaturated fatty acid oleic acid protects *E*. *faecalis* against acute membrane stress caused by bile salts [30]. More recently, exogenous fatty acids were shown to confer protection against membrane stress caused by DAP despite the absence of LiaR, one of three components in the antibiotic-response regulator LiaFSR [31].

In recent years, several studies investigating enterococcal lipids in the context of resistance to antibiotics and antimicrobial peptides have been reported. The emergence of DAP resistance in enterococci has been correlated with lower fluidity and lower PG content and higher glycerophospho-diglucosyl-diacylglycerol (GPDGDAG) content. Total lysyl-PG (LPG) levels were unchanged. [3]. Lysyl-PG is a product of the bifunctional integral membrane enzyme known as multiple peptide resistance factor (MprF), which modifies PG with positively charged amino acids such as lysine in the inner CM leaflet, and then translocates or “flips” lysyl-PG to the outer leaflet. This modification mediates resistance to cationic antimicrobial peptides (CAMPs) such as colistin, nisin, human β-defensin 3 (hBD-3) and polymyxin B [22, 32–34]. DAP interacts with the septa of DAP-sensitive *E*. *faecalis*; however, changes in lipid composition and distribution in DAP-resistant strains are associated with changes in DAP interactions that move it away from the division septum to other cell membrane regions [6]. Thus, environmental factors and antimicrobial compounds exert considerable influences on lipid composition.

Bacterial cardiolipins (CLs) are compartmentalized into distinct membrane domains that play a role in cell division and membrane transport through lipid-protein interactions [35]. The first bacterial lipid domains reported were CL domains in *B*. *subtilis* [11, 36] and *E*. *coli* [11, 35, 37, 38]. CL domains are likely formed by the high negative curvature strain that is caused by CL in the lipid bilayer, owing to relative difference in size between its head group and its much larger hydrophobic domain [35].

To understand precisely how different lipid compositions contribute to antimicrobial resistance, we have performed a mass spectrometric (MS) analysis of the lipid composition of *E*. *faecalis* for the purpose of correlating individual lipid species with antibiotic resistance. Previous studies obtained the phospholipid content of daptomycin-resistant enterococci first by thin-layer chromatography (TLC) and then confirmation of TLC-separated products by tandem MS [3, 6] or solely by TLC [32]. These studies did not report the identities of individual lipid species. Here we have deployed MS-based methods directly on Enterococcal lipid extracts, and provide a detailed characterization of lipid species at the molecular level Our methods allow us to forgo TLC and analyze extracted lipids directly via MS, enabling the broadest possible analysis of lipids across various lipid classes. By taking advantage of high resolutions and sensitivities, we can obtain a snapshot of phospholipids and glycolipids that *E*. *faecalis* needs for growth and adaptation to antimicrobial challenge. Taking our lipidomic characterization one step further, we have employed the widely used approach of multiple reaction monitoring (MRM) for quantification of *E*. *faecalis* lipids. Using state-of-the-art MS platforms, our methods have better sensitivity, selectivity and accuracy, and can be used both for untargeted and targeted lipid analyses and rapid quantification. We have identified individual species of phosphatidylglycerols (PG), lysyl-PG (L-PG), cardiolipins (CL), glycerophospho-diglucosyl-diacylglycerols (GPDGDAG), diglucosyl-diacylglycerols (DGDAG), diacylglycerols (DAGs), and triacylglycerols (TAGs). Given the important role played by fatty acids in stress responses, we report fatty acid compositions for the lipids that we have identified. Furthermore, we have measured the concentrations of several PG species in *E*. *faecalis* OG1RF throughout the growth curve, and show that PG levels vary substantially as cells progress through the logarithmic phase to the late stationary phase. We also show that a subset of lysyl-PGs and PGs are substantially lower in two DAP-resistant (DapR) strains compared to DAP-sensitive wild type. The data and technical information that we provide in this study will lay the foundation for future lipidomics studies in *E*. *faecalis* and other bacteria, and provides a more detailed understanding of the lipidomic basis of DAP resistance.

## Materials and methods

### Bacterial strains and cultures

*Enterococcus faecalis* strains OG1RF [39], Dap21 and Dap22 were inoculated from single colonies grown on Brain Heart Infusion (BHI) agar at 37°C into BHI broth and was grown statically overnight for 15–18 h for all assays except the *in vitro* growth phase study. In addition, for the growth phase study, OG1RF was inoculated into BHI broth and grown statically until optical densities of 0.3 (early-logarithmic phase), 0.5 (mid-logarithmic phase), 0.8 (late-logarithmic phase) and 1.3 (late stationary phase) were achieved. Broth cultures were centrifuged, the cell pellets washed once with 1X PBS, and centrifuged again to harvest the cell pellets. Cell pellets were dried overnight on a lyophilizer and the dry cell pellets weighed.

### In vitro evolution against DAP

The *in vitro* evolution protocol used here for *E*. *faecalis* OG1RF was adapted from that of *E*. *faecalis* V583 [40]. For each parent strain, multiple parallel lines of evolution were performed. The evolution experiment started 1:10 dilutions of overnight bacterial cultures into fresh BHI broth supplemented with calcium at 1.25 mM, and containing DAP at concentrations equivalent of the MIC, 2xMIC and 4xMIC. After 22 to 26-hour static incubation in 2 mL Eppendorf tubes at 37°C, cultures of each line were examined for visible bacterial growth. Bacterial cultures at the highest growth-permissive concentrations (HGPCs) were diluted 10 times into fresh DAP-containing medium at 0.5xHGPC, HGPC and 2xHGPC. We repeated the same incubation and dilution steps until the HGPC reached 512 μg/mL. Bacterial cultures were then passaged in plain BHI broth for 3 days, followed by a repeat test of the MIC to ensure the mutants were stably resistant to DAP. Passaged cultures were also streaked on selective media containing 25 μg/mL rifampicin (Sigma) plates and CHROMagar^™^ Orientation plates to ensure the absence of contamination by other microbes.

### Whole-genome sequencing

Genomic DNA was extracted from overnight bacterial culture using PureLink Genomic DNA Mini Kit (Thermo Fisher Scientific). The samples were then sequenced on an Illumina MiSeq v3 platform. We analyzed the genomic sequence using CLC Genomics Workbench 8.0. The complete OG1RF reference genome (NC_017316) from NCBI database was used for mapping and annotation. The threshold variant frequency was set as 35%. Non-synonymous mutations within coding regions were filtered out. All structural variations were manually confirmed on the mapping track.

### Daptomycin Minimal Inhibitory Concentration (MIC) with broth microdilution

The broth microdilution method was adapted from a published protocol [41]. Daptomycin (AG Scientific) was dissolved in ultrapure water and sterilized by filtration. The DAP stock solution was then further diluted in Brain Heart Infusion (BHI) broth (Acumedia, Neogen) supplemented with 50 mg/L Ca^2+^. Mid-log phase bacteria were diluted to 5 × 10^5^ CFU/mL in a 96-well plate containing serial 2-fold dilutions of DAP or in the absence of DAP as a control. Technical triplicates were performed in each of 3 biological replicates. After overnight incubation at 37°C, wells were visually examined for presence of bacterial growth. The lowest DAP concentration with no visible bacterial growth was defined as the MIC. All incubations were static. The standard Mueller Hinton Broth (MHB) was not used because it significantly slows growth of *E*. *faecalis* OG1RF (data not shown).

### Lipid extraction

Lipids were extracted from OG1RF cell pellets using a modified Bligh & Dyer method in which the extraction solvent contained chloroform/methanol in a ratio of 1:2 (v/v). For method validation, samples were spiked with known amounts of internal standards. 900 μl of chilled extraction solvent containing internal standards (purchased from Avanti polar lipids, Alabaster, AL, USA)) was added to the cell pellets (Table 1):

**Table 1:** Internal standards that were spiked into *E*. *faecalis* lipid extracts.

Internal standard	Catalogue #	Stock conc. [mg/ml]	Final conc. [μg/ml]	Precursor ion (m1) *m/z*	Fragment ion (m3) *m/z*
PG 14:0	840445P	1	5	665.5 [M-H]^-^	153
Lysyl PG 16:0	840520P	0.1	4	849.6 [M-H]^-^	145
CL 14:0	750332P	1	100	1239.9 [M-H]^-^, 619.5 [M-2H]^2-^	153, 227

The suspension was vortexed for 15 seconds and incubated with agitation on a thermomixer at 1000 rpm at 4°C in the dark for 1 hour. Following a quick spin, 300 μl chilled chloroform and 250 μl chilled deionized water were added to the suspension, which was vortexed for 15 seconds. The suspension was centrifuged at 9,000 rpm for 2 min to achieve separation into aqueous and organic phases. The organic phase (containing lipid) was transferred into a clean 1.7 ml centrifuge tube. 500 μl of chilled chloroform was added to the remaining aqueous phase and residual lipids extracted by repeating the above steps. The first and second organic extracts were pooled together and were subsequently centrifuged in a vacuum concentrator (SpeedVac) until they were dry. The dried lipid extract was resuspended in a mixture of chloroform and methanol (1:1 v/v), to achieve a final lipid concentration of 10 mg/ml. This solution was stored at -80°C until the mass spectrometry analysis was performed.

### Mass spectrometry

As a first approach, the lipid profile of *E*. *Faecalis* OG1RF was obtained through the use of high-resolution mass spectrometry. The OG1RF lipid extract was diluted 2:1 (v/v) with solvent containing isopropanol:methanol:chloroform (4:2:1, v/v) and 2 mM ammonium acetate. 10 μl of each sample was injected into an LTQ-Orbitrap XL mass spectrometer (Thermo Fisher Scientific, USA) using the Advion Nanomate interface. The nanospray voltage applied was −1.35kV in negative ionization mode. Mass spectra were acquired at a resolution of 30,000 and 100,000. Product ion scans (PIS) involving collision energies between 35 and 45 eV were used to identify α-alkyl chains.

Diacylglycerols (DAGs) and triacylglycerols (TAGs) analysis was undertaken by liquid chromatography coupled to mass spectrometry (LC-MS) with electrospray ionization (ESI) on an LTQ-Orbitrap XL (Thermo scientific, USA). A Zorbax Eclipse XDB-C18 column (3.0x150 mm, 1.8μm particle size, Agilent Technologies, USA) and a mobile phase consisting of a mixture of isopropanol:methanol:chloroform (4:2:1, v/v) and 2 mM ammonium acetate were used to separate lipids TAG from more polar lipids (DAG and glycerophospholipids). The separation was done isocratically at a flow rate of 130 μl/min. The total runtime was 30min. The LTQ-Orbitrap XL was operated with positive ionization at an electrospray voltage of 3.5 kV and mass-to-charge *(m/z)* range of 300–1400. Under these conditions, the TAG ionize as ammonium adducts and elute between 8 and 19min depending on chain length and unsaturation.

Phosphatidylglycerol (PG) and lysyl-phosphatidylglycerol (L-PG) in *E*. *faecalis* were quantified by LC-MSMS using multiple reaction monitoring (MRM). Lipid internal standards were added to the sample prior to lipid extraction. An Agilent 6460 QqQ mass spectrometer connected to a 1290 series chromatographic system was used. ESI was used to ionize lipids. Each lipid molecular species was analyzed using a targeted multiple reaction monitoring (MRM) approach containing transitions for known precursor/product mass-to-charge ratio (m1/m3) [42]. The experimental conditions and the MRM transitions required for lipid quantification are reported in the supplement.

Cardiolipins (CL) analysis was undertaken using the Agilent 6550 Q-ToF mass spectrometer connected to a 1200 series HPLC-Chip HILIC chip system. A customized HILIC-chip containing Amide-80 stationary phase (5 μm particle size, 80 Å pore size, Tosoh Bioscience, LLC. Montgomeryville, PA) was also prepared, along with a trapping column (160 nl) and an analytical column (75 μm × 150 mm) (Agilent Technologies Corp., Santa Clara CA). The solvents used for HPLC with the HILIC column were: 50% acetonitrile and 50% water containing 25 mM ammonium formate pH 4.6 (solvent A) and 95% acetonitrile and 50% water containing 25 mM ammonium formate pH 4.6. The pH value was adjusted with formic acid. Analytes spiked with internal standard were eluted with the following gradient: 100% solvent B from 0 to 1.5 minutes, 40% solvent B from 1.5 to 8.5 minutes, 30% solvent B from 8.5 to 10.5 minutes, 0% solvent B from 11.5 to 13.0 minutes, 100% solvent B from 13.1 to 19 minutes. The chip cube was operated with back flush mode. Each sample was injected through the enrichment column at a flow rate of 4 μl/min. 1.5 min after injection, the valve was switched to align the analytical column with the enrichment column at a flow rate of 400 nl/min. The QToF instrument was set to the negative ion mode, with an electrospray voltage of -1580 V (Vcap), a temperature of 185°C, a drying gas rate of 4 (or 12) l/min, and a fragmentor voltage of 150 V. Spectra were acquired in the targeted tandem MS mode. The MS acquisition rate was 2 spectra/sec and the tandem MS acquisition rate was 2 spectra/sec. Data are reported for 3–5 biological replicates.

### Data processing

High-resolution MS spectra obtained on the LTQ-Orbitrap XL were processed using Xcalibur software (Thermo Fisher Scientific, USA). Lipid species were identified using an in-house database as well as online LipidMaps and Metlin lipid databases with an MS tolerance of 10 ppm. The identification was based on accurate mass and/or retention times (RT). Tandem MS data obtained on the Agilent 6550 Q-ToF were analyzed to obtain headgroup and/or fatty acid compositions and the RT for each lipid molecular species. For MRM data obtained on the Agilent 6460 QqQ, signal intensities were compared with the intensities from the spiked internal standards (PG 14:0 for PG and Lysyl-PG 16:0 for Lysyl-PG) and the retention times for the various classes were matched. Growth-phase MRM data were normalized to the dry cell pellet weights to ensure that comparisons were made per unit weight of bacterial sample. For the growth curve study, MRM peak areas for the early-logarithmic, mid-logarithmic and late-logarithmic phases were normalized to the peak areas for the late stationary phase to obtain a fold change. These fold changes were used to generate a heat map in R. The Student’s t test was performed to determine whether differences between OG1RF, Dap21 and Dap22 strains were statistically significant. p <0.05 was considered statistically significant.

## Results

### Identification of phospholipids: Phosphatidylglycerol (PG), Lysyl-PG (LPG) and Cardiolipin (CL)

**Fig 1:**
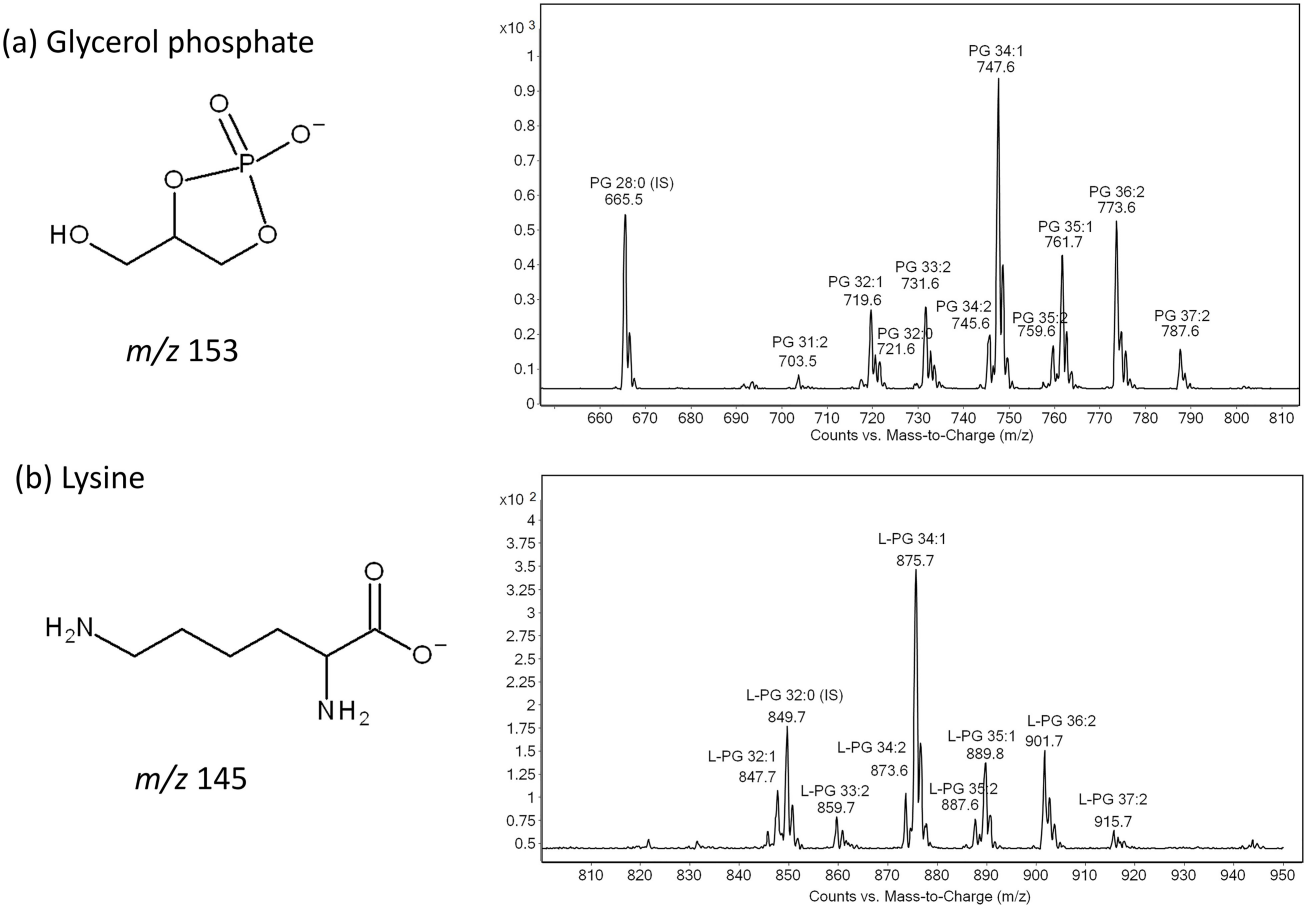
Precursor ion scans for *Enterococcus faecalis* phospholipids. Precursor ion scans for (A) 153 fragment ion (phosphatidylglycerol (PG))) and (B) 145 fragment ion (lysyl-phosphatidylglycerol (LPG)) reveal several species of PG (A) and of LPG (B). Each peak is labelled with the class (PG or LPG), fatty acid composition (e.g. 34:1), and the mass-to-charge *(m/z)* ratio (e.g. 747.6 for PG 34:1).

Anionic phosphatidylglycerol (PG) and cardiolipins (CL) are the major phospholipids in Gram-positive bacteria. In addition to PG and CL, Gram-negative bacteria contain zwitterionic phosphatidylethanolamine (PE) in abundance [13]. Based on this information, we performed a precursor ion scan (pre-IS) for the glycerol phosphate headgroup (*m/z* = 153) of PG on the Gram-positive bacterium, *E*. *faecalis*, and observed a number of peaks corresponding to PG (Fig 1a). The 665.5 peak corresponds to DMPG, the PG internal standard, whereas the endogenous *E*. *faecalis* peaks are seen at 703.5, 719.6, 721.6, 745.6, 747.6, 759.6, 761.6, 773.6, and 787.6.

Since *E*. *faecalis* is known to produce the aminoacylated PG, lysyl-PG (LPG), we also performed a pre-IS for the lysine moiety (*m/z* = 145). In addition to the peak (849.7) corresponding to the LPG internal standard, lysyl-DPPG, we observed peaks at 847.7, 859.7, 875.7, 873.7, 887.6, 889.8, 901.7, and 915.7 (Fig 1b). Based on these data, we conclude that only a subset of PGs, i.e. PG 32:1, 33:2, 34:1, 34:2, 35:1, 35:2, 36:2 and 37:2, serve as substrates for the MprF enzyme that trans-esterifies amino acids to PG [43–45].

**Fig 2:**
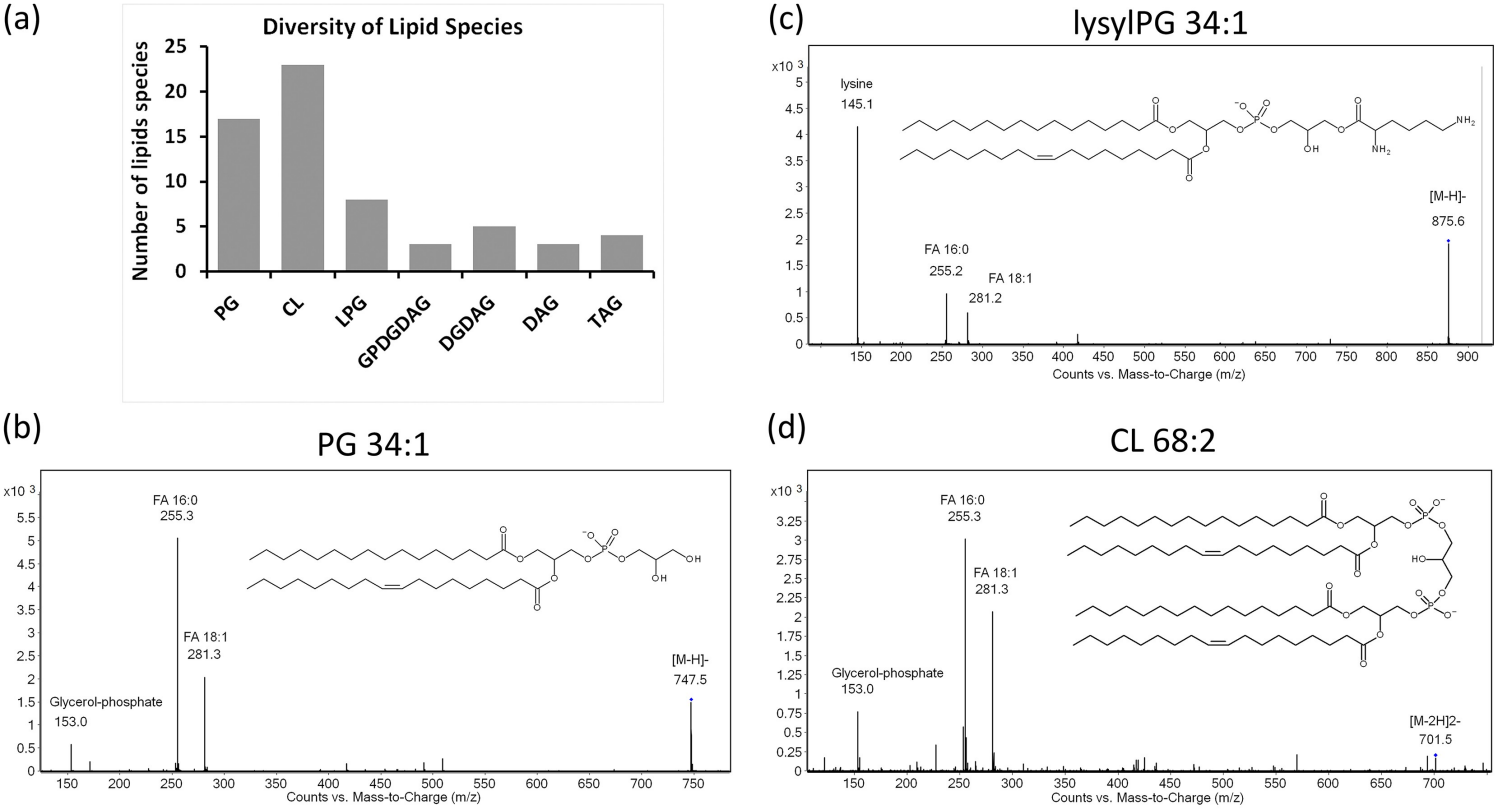
The diversity of lipid species in E. faecalis strain OG1RF. (a) The total number of lipid species identified is 63 across 7 classes. Representative MSMS spectra for the phospholipids (PG, lysyl-PG, and cardiolipin (CL)) are shown for (b) PG 34:1, (c) lysyl-PG 34:1, and (d) CL 68:2. Each species is identified based upon its characteristic fragmentation pattern: MSMS of (b) PG 34:1 gives rise the glycerol-phosphate headgroup (m/z = 153) and fatty acids 16:0 (m/z = 255.3) and 18:1 (m/z = 281.3); (c) lysyl-PG 34:1 gives rise to lysine (m/z = 145) and fatty acids 16:0 and 18:1; and CL (68:2) gives rise to the glycerol-phosphate headgroup and fatty acids 16:0 and 18:1.

For a more detailed characterization of phosphatidylglycerol (PG) species in *E*. *faecalis*, we performed LC-MS on membrane lipids to achieve separation of lipid species according to class followed by species identification. and observed eight additional PGs, twenty three cardiolipins (CLs), three glycerophospho-diglucosyl-diacylglycerols (GPDGDAGs), five diglucosyl-diacylglycerols (DGDAGs), four diacylglycerols (DAGs), and four triacylglycerols (TAGs) (Fig 2a and S1–S3 Tables). Lipids of a given class (e.g. PG or LPG) differ from each other in the number of carbon atoms per fatty acid chain and the number of carbon-to-carbon double bonds in unsaturated chains. For example, PG 34:1 contains 34 carbon atoms in its two fatty acyl chains and one of these chains contains a single double bond (Fig 2b). Fig 2c shows the structure of LPG 34:1 which contains the same number of fatty acyl carbon atoms and double bonds as PG 34:1, but is conjugated to a lysine amino acid, which is characteristic of the LPG class. The structure of CL 68:2, shown in Fig 2d, is representative of the cardiolipin or di-PG class of phospholipids and contains twice as many carbon atoms and double bonds as PG 34:1.

The CL species appeared predominantly as [M-2H]^2-^ ions (Fig 2d and S2 Table). The ionization of CL is more complex than that of PG because of CL’s larger structure [46]. The fact that the [M-2H]^2-^ ions produce a characteristic signature within the MS spectrum in which the isotopic peaks are separated by a mass-to-charge *(m/z)* ratio of 0.5 [47] allowed us to identify CLs here (S1b Table). The number of carbon atoms in CL fatty acyl chains ranges from 64 to 70, and the degree of unsaturation ranges from 1 to 4 double bonds. Thus we see that the carbon atom number and degree of unsaturation of CLs is roughly twice that of PGs, which is consistent with PG being the substrate of cardiolipin synthase (Cls) 1 or 2.

Finally, using the same methodology, we detected three species of the phospholipid glycerophospho-diglucosyl-diacylglycerol (GPDGDAG) (Fig 2a and S1 Table). Tandem MS data from a previous study on GPDGDAG in *Streptococcus mutans* [48] guided us in the identification of three species of GPDGDAG in *E*. *faecalis* OG1RF. Although it is not a major bilayer-forming lipid, higher levels of GPDGDAG were nonetheless associated with daptomycin resistance in *E*. *faecalis* and *E*. *faecium* [3].

### Identification of glycerolipids: Diacylglycerol (DAG) and Triacylglycerol (TAG) species

The synthesis of lipoteichoic acids (LTA) and membrane-derived oligosaccharides in bacteria leads to the production of diacylglycerols (DAGs) as secondary products [49]. DAG is a neutral lipid whose accumulation in the membrane leads to bilayer disruption [50]. For this reason, it is metabolized to form phosphatidic acid and other phospholipids [9, 49]. Little has been reported about bacterial triacylglycerol (TAG) [51], but its main function is as a reservoir of fatty acids [49]. TAG can also regulate membrane fluidity and serve as an electron sink [9]. We have identified three species of diacylglycerol (DAG) and four species of TAG in *E*. *faecalis* (S3 Table). As DAG and TAG do not ionize well on their own, a charged group or adduct needs to be attached to the parent DAG or TAG molecule. Therefore, for all species, we detected the positive ammonium adduct—[M+NH_4_]^+^ (S1 Fig and S3 Table). In addition, we have identified five species of diglucosyl-diacylglycerol (DGDAG) (Fig 2 and S1 Table). The DGDAG species were detected as negative formate ([M+HCOO]^-^) adducts.

### Tracking PG and lysyl-PG levels during *in vitro* growth

**Fig 3:**
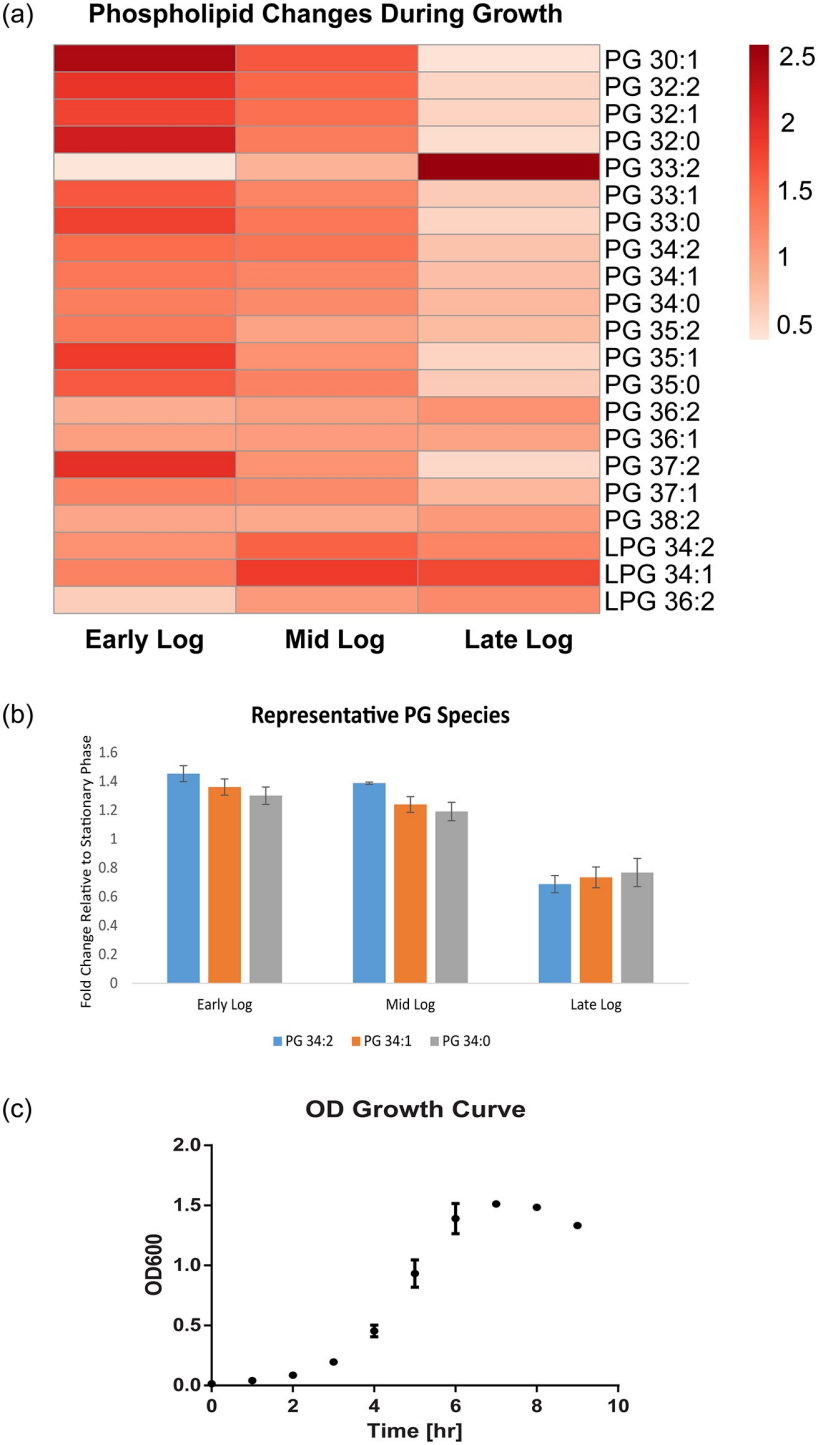
The levels of *E*. *faecalis* strain OG1RF PG and LPG can be tracked throughout the growth curve. (A) Heat map depicting phosphatidylglycerol (PG) and lysyl-PG levels in the early-, middle- and late-logarithmic growth phases relative to the late stationary phase. The colour scale (top right) indicates fold change of phospholipids (PG and lysyl-PG) in early-, mid-, and late-logarithmic phase cells with respect to late stationary phase cells. (B) Bar chart showing representative fold change values for PG 34:0, 34:1 and 34:2. (C) The three logarithmic phases corresponded to optical density (OD) values of 0.3, 0.5 and 0.8 on an OD growth curve.

The quantification of *E*. *faecalis* phospholipids to obtain lipid concentrations via the mass spectrometry (MS) scanning mode known as multiple reaction monitoring (MRM) has not been previously reported. Earlier reports of enterococcal phospholipid quantification relied on spectrophotometric methods applied to excised TLC plate spots [3, 6]. Here, we have used MRM for direct quantification and tracked the levels of seventeen PG species during the *in vitro* growth of *E*. *faecalis*. We performed MRM on samples extracted during the exponential (early-logarithmic, mid-logarithmic, late-logarithmic) (Fig 3a) and late stationary (overnight) growth phases and quantified PG and lysyl-PG levels. In MRM, the *m/z* values of both the precursor and fragment ions are specified. Quadrupole Q1 isolates precursor ions, quadrupole Q2 fragments the precursor ions, and quadrupole Q3 allows selected fragment ions to pass through to the detector, which measures the abundance of each fragment [52]. The *m/z* of the precursor ion and the *m/z* of the fragment ion constitute one “MRM transition”, and each transition gives rise to a peak on the ion chromatogram. MRM transitions for *E*. *faecalis* PGs and LPGs are listed in S4 Table. Integrating a peak gives the peak area, which is proportional to the abundance of a single lipid species (e.g., PG 34:1). Dividing each peak area by the peak area of the internal standard gives a lipid concentration (μg/ml), and further dividing by the dry weight of the cell pellet gives a normalized lipid concentration. We further divided each normalized lipid concentration from early-log, mid-log or late-log phase by the corresponding late stationary normalized lipid concentration to obtain a fold change. We used these fold change values to generate the heat map in Fig 3a. Using this analysis protocol, we uncovered measurable differences in the levels of PGs and LPGs at different growth stages (Fig 3a). The short-chain PGs 30:1, 32:2, 32:1 and 32:0 follow similar trends in that they are abundant in early log phase and decrease through mid log phase to late log phase. PG 33:2 levels were unlike the other PGs in that it was low in early log phase and very high in late log phase. This stark difference between PG 33:2 and the more saturated PG 33:1 and 33:0 suggests that the two double bonds in PG 33:2 may be important for growth. Levels of PG 34:0, 34:1 and 34:2, which are also depicted in graphical form in Fig 3b, decreased throughout the exponential growth curve. There are slight differences in the late-log phase concentrations of PG 35:0, 35:1 and 35:2, but they otherwise also show a downward trend from early to late log phase. PG 36:1, PG 36:2 and PG 38:2 levels remain almost constant throughout the growth curve. The trend for PG 37:2 almost mirrors that of PG 33:2:PG 37:2 is highest in early log phase and lowest in late log phase. PG 37:1, which has one fewer double bond than PG 37:2, remains constant in early and mid log phase but decreases slightly in late log phase. The overall trend for PGs is that their amounts decrease during exponential growth to a certain minimum level in late-log phase, and then increase to a level above this minimum as they progress into stationary phase. The overall trend for the LPGs is opposite to that of the PGs. LPG levels tend to increase throughout the growth curve, suggesting that *E*. *faecalis* may fortify its membrane with positively-charged phospholipids as it propagates. As the bacteria divide exponentially, they may need to maintain a certain optimum ratio of anionic PG and cationic LPG in order to ensure that various membrane-dependent processes continue to function optimally under the prevailing growth conditions.

### Phospholipid levels in daptomycin-resistant strains

In order to study the lipid profiles of antibiotic-resistant *E*. *faecalis* strains, we generated two daptomycin-resistant (DapR) strains via *in vitro* evolution from the DAP-sensitive OG1RF strain. We performed whole genome sequencing to identify the single nucleotide polymorphisms (SNPs) in these DapR strains (Table 2). Dap21 harbours mutations in three different genes: cardiolipin synthase 1 (*cls1*; RF10364), a gene encoding a putative chaperone protein (RF11464), and a gene encoding hypothetical membrane protein (RF11507). Dap22 harbours mutations in the same chaperone- and membrane protein-encoding genes that are mutated in Dap21, as well as mutations in cardiolipin synthase 2 (*cls2*; RF11324) and a putative metal-dependent HD-domain-containing hydrolase (RF11901).

**Table 2:** Single Nucleotide Polymorphisms (SNPs) in the DapR strains Dap21 and Dap22.

Strain		Gene/locus				
		*cls1* (OG1RF_10364)	*cls2* (OG1RF_11324)	OG1RF_11464	OG1RF_11507	OG1RF_11901
Dap21	Mutation type	Substitution	-	Frameshift	Frameshift	-
	Mutation location	R267C	-	272	19	-
Dap22	Mutation type	-	Substitution	Frameshift	Nonsense	Inframe insertion
	Mutation location	-	P181H	272	L4	84TARA85

The minimum inhibitory concentrations (MICs) of DAP for both of these DapR strains are significantly higher than the MIC for OG1RF (Table 3).

**Table 3:** Minimum Inhibitory Concentrations (MICs) of daptomycin for three *E*. *faecalis* strains.

Strain	MIC [μg/ml]
OG1RF	1–2
Dap21	128
Dap22	128

**Fig 4:**
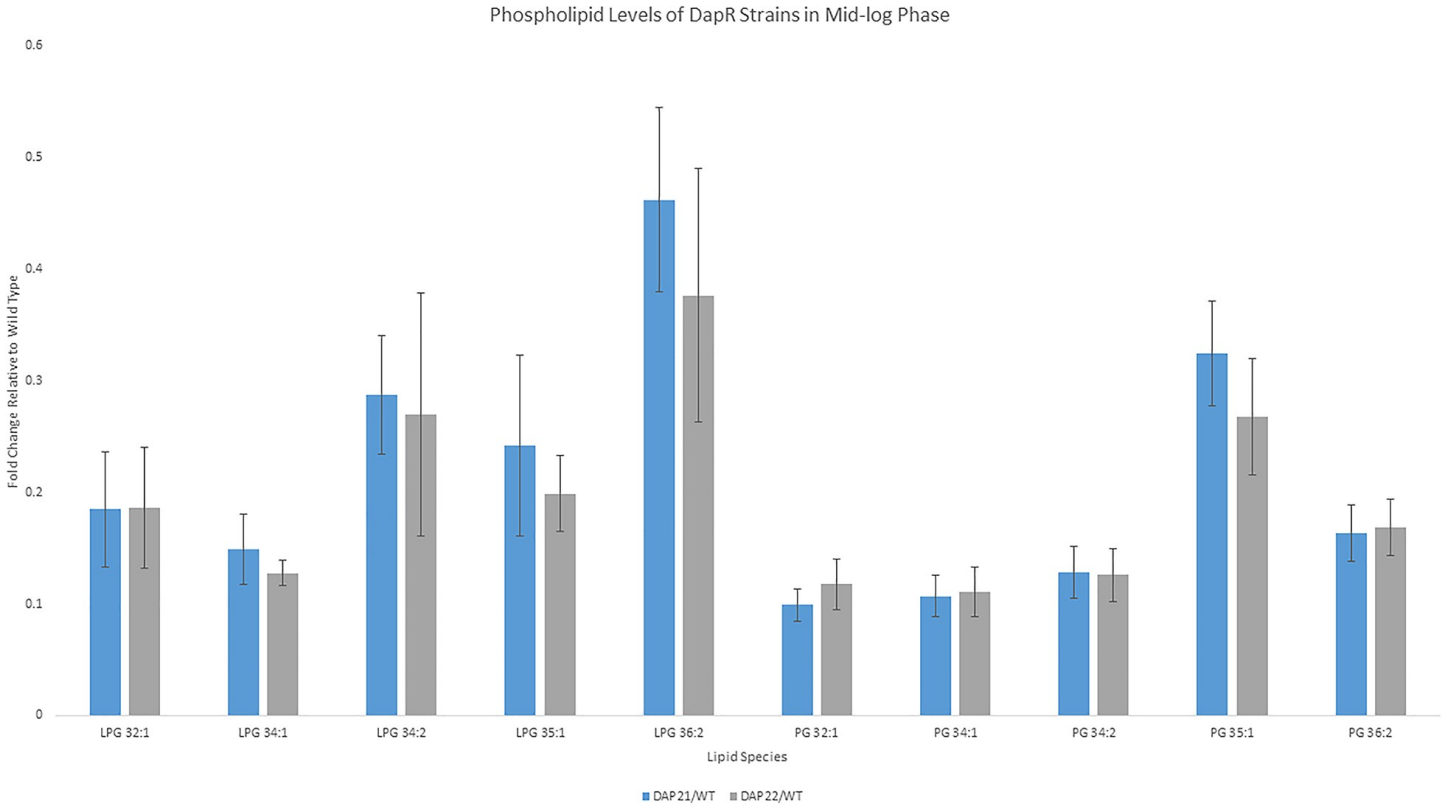
The levels of PG and LPG in two daptomycin-resistant strains, Dap21 to Dap22, compared to non-resistant OG1RF. The bar chart shows the fold change of 5 LPGs and their corresponding PGs from Dap21 and Dap22 grown to mid-logarithmic phase, with respect to the non-resistant OG1RF parental strain. Data are expressed as mean ± standard deviation (SD) (calculated from 5 replicates) with the SDs represented as error bars.

We quantified PG and LPG levels in Dap21 and Dap22, as compared to wild type, and observed that that at mid-log phase (OD = 0.5), levels of 5 LPGs and the corresponding PGs were lower in both DapR strains than in OG1RF (Fig 4). The differences in PG and LPG levels between OG1RF and Dap21, and between OG1RF and Dap22, are all statistically significant, with all p values being less than 0.05. The phospholipid profiles of Dap21 and Dap22 do not differ significantly from each other another, however, suggesting that the emergence of DAP resistance is associated with an overall decrease in LPG and PG levels.

## Discussion & conclusion

There is great diversity in lipid composition across bacterial species, and environmental conditions can affect the lipid composition of a single species [10]. Membrane lipid homeostasis and adaptation to changing environmental conditions are essential for bacterial survival. Variations may occur in response to temperature, pH, osmolarity, salinity and the presence of chemical compounds [8]. In response to these changes, bacteria modify membrane-forming phospholipids by altering specific properties of their fatty acyl chains, e.g. changing the degree of unsaturation (i.e. the number of double bonds), adopting either a *cis* or *trans* configuration for unsaturated chains, adjusting the ratio of *iso* to *anteiso* chains, or introducing a cyclopropane ring [8]. Lipid changes affect membrane proteins whose functions can be affected in two main ways: 1) by the chemical properties of lipids, e.g. length of fatty acid chains, number of carbon-carbon double bonds, nature of the head group, net charge and chelation; 2) by the ability of lipids to associate with themselves as a result of bulk properties, e.g. molecular shape, viscosity/fluidity, molecular packing and thickness of the lipid bilayer [53]. Hence, to fully appreciate lipid-protein interactions in the membrane, we need information about lipids as well. Lipidomics is thus essential to understanding bacterial physiology.

Besides these physiological roles for lipids, changes in bacterial lipid composition have been correlated with antibiotic resistance. In 1970, Dunnick and O’Leary showed that for Gram-negative bacterial strains resistant to polymyxin, tetracyclines and penicillin, the proportion of fatty acids containing cyclopropane was lower than that in sensitive strains [54]. More recently, a drop in PG levels was correlated with DAP resistance in *E*. *faecalis* and *E*. *faecium* [3]. In *E*. *faecalis*, DAP resistance was also related to mutations in genes involved in lipid metabolism, namely, glycerophosphoryldiester-phosphodiesterase *(gdpD)*, cardiolipin synthetase *(cls)*. Multiple peptide resistance factor *(mprF)* in *Staphylococcus aureus* and enterococci has also been associated with DAP-resistance [5, 40, 55–58]. The comprehensive lipidomic analysis that we report here reveals that DAP resistance in *E*. *faecalis* is accompanied by a decrease in both lysyl-PG and PG levels in strains that harbour a single point mutation in a cardiolipin synthase. Since DAP is known to bind to PG, we would expect lower PG levels to confer resistance to DAP, and our results support this notion. On the other hand, the reduction in lysyl-PG is counterintuitive since this cationic phosphospholipid would help repel cationic DAP from the cell membrane. However, *E*. *faecalis* strains harbouring deletions in genes encoding multiple peptide resistance factor (*mprF*), the enzyme that synthesizes LPG, show a minimal change in the DAP MIC and suggesting that lysyl-PG alone does not determine DAP susceptibility [32].

The compartmentalization of bacterial lipids into discrete membrane domains is also of growing interest and is likely to depend on both the chemical and bulk properties of lipids [59, 60]. These domains include PG domains, CL domains, and lipid rafts [12–15, 35, 61]. These domains coordinate a range of processes, including growth, cell division, and antibiotic resistance [11, 12, 17]. One example of a domain-specific process is the secretion and biogenesis of cell-surface proteins [18, 62]. In *E*. *faecalis*, these proteins include virulence factors including pili, which are secreted via the general secretion (Sec) machinery, assembled by sortase C, and attached by sortase A to the cell wall within focal domains [18, 22]. Extracellular secretion is essential to bacterial pathogenesis because it allows bacteria to release virulence factors and toxins into the host [63]. In *E*. *faecalis*, virulence factors that are assembled in distinct domains include pili and aggregation substance, both of which contribute to disease progression [64–67]. In *B*. *subtilis*, SecA becomes delocalized when anionic PG are depleted [19], while in *S*. *pyogenes*, the focal localization of a protease called HtrA is physically linked to domains containing anionic lipids [20]. In *B*. *subtilis*, *and S*. *aureus*, lipid rafts play roles in sporulation, cell division, secretion and biofilm formation, and can also serve as drug targets [68–71]. To better understand the role of lipids in the physiology, pathogenesis and antibiotic resistance of *E*. *faecalis*, we must begin with a detailed characterization of its lipids. The methods we report here could be used to study the lipid composition of *E*. *faecalis* membrane domains. Since DAP is known to bind focally to *E. faecalis [6]*, MRM-based quantification of phospholipids within these target domains would reveal what quantities of phospholipids confer resistance to DAP.

Previous studies characterizing the lipids of *E*. *faecalis* were performed by thin layer chromatography and subsequent MS of lipid spots, and revealed several classes of lipid molecules including phosphatidylglycerol (PG), cardiolipins (CL), glycerophospho-diglucosyl-diacylglycerol (GPDGDAG), and aminoacyl-PG [6, 43, 72, 73]. Here, using liquid-chromatography mass spectrometry (LC-MS), we have detected previously described lipids, i.e. PG, LPG, GPDGDAG, and identified previously unidentified *E*. *faecalis* lipids including DGDAG, DAG and TAG. Previous reports on *E*. *faecalis* lipids are summarized in Table 4 and are compared against the findings achieved in the present study. The methodology we report here provides high selectivity, sensitivity and accuracy and enabled us to identify several PG and CL species in *E*. *faecalis* strain OG1RF by accurate mass determination and tandem MS fragmentation analysis. We provide the most detailed analysis of *E*. *faecalis* lipids performed to date.

**Table 4:** Previous biochemical analyses of *Enterococcus faecalis* compared to the present study.

	Bao et al. (2012)	Mishra et al. (2012)	Tran et al. (2013)	Fozo et al. (2014)	This Study
Growth phase	Late stationary	Late stationary	Late stationary	Late stationary	Exponential, Late stationary
GPL					
PG	+	+	+	-	+
CL	+	+	+	-	+
Lys-PG	-	+	+	-	+
Glycerolipids					
DAG	-	-	-	-	+
TAG	-	-	-	-	+
DGDAG	+	-	-	-	+
GPDGDAG	-	+	+	-	+
Fatty acids	-	+	-	+	+ (acyl chains by MSMS)
Methodology	TLC & molybdenum, ninhydrin staining	TLC, GC-FAME & ESI-MSMS	TLC, ESI-MSMS	GC-FAME	TLC, ESI-MSMS
Resolution of molecular species	-	-	-	-	+
Quantification	-	-	-	-	+
Number of molecular species quantified by MRM	0	0	0	0	20

In bacteria, membrane lipid biosynthesis begins with fatty acid synthesis via the type II fatty acid biosynthetic pathway (FASII), and is followed by the transfer of fatty acid chains to glycerol-3-phosphate in the membrane to form phosphatidic acid (PA). PA is the common precursor to PG, LPG and CL [8]. GPDGDAG is one of the glycolipid precursors of lipoteichoic acid (LTA), a component of the Gram-positive cell envelope [3]. Another component of the Gram-positive cell envelope is DGDAG, which is formed when a uridine diphosphate sugar reacts with DAG [74]. Both DGDAG and MGDAG serve as membrane anchors for LTA in the cell envelope of many Gram-positive bacteria [75]. DAG is formed during phospholipid turnover, while TAG is formed from DAG by the enzyme diacylglycerol acyltransferase [8, 51]. We quantified the PG and LPG species by multiple reaction monitoring (MRM). The lipid GPDGDAG is important because daptomycin-resistant strains of *E*. *faecium* and *E*. *faecalis* were reported to contain a higher GPDGDAG concentration [3]. In a daptomycin-resistant (DapR) clinical isolate of *E*. *faecalis*, CL was re-distributed away from the septum but total CL levels remain unchanged [6]. The same DapR isolate had lower levels of PG [3]. LPG confers resistance to *E*. *faecalis* against the antimicrobial peptide human β-defensin 2 (hBD2) [22, 32]. In contrast to previous studies on DapR *E*. *faecalis* strains that showed LPG levels were unchanged, highly sensitive MRM analysis used here reveals that LPG levels are lower in the two DapR strains that we generated from a susceptible parental strain via *in vitro* evolution. One result of decreased LPG synthesis might be that more of the PG substrate can be consumed in an alternative pathway, e.g CL and GPDGDAG biosynthesis.

Mass spectrometric identification of cardiolipin has been challenging due to its complex structure and complex ionization [46]. The cardiolipin structure is complex because it contains a polyglycerophosphate backbone connected to four acyl chains [76], which is twice as large as a PG molecule with only two acyl chains connected to one glycerophosphate backbone. Historically, CLs as a class were studied using radiochemical labelling [77] or another method that coupled high-performance liquid chromatography (HPLC) to fluorescence detection [78]. Fatty acid analysis of the total CL pool was then carried out [79, 80]. Since individual CL molecular species were not directly analyzed, these methods could not determine the number of different CL species present. Despite the technical challenges, we were able to identify the [M-2H]^2-^ ions of CL species by LC-MS. CLs have been reported to predominantly form [M-2H]^2-^ ions [81] and we thus searched for these doubly charged ions in our own spectra, where we observed a [M-2H]^2-^ ion “signature”. CL mass spectrometry studies are ongoing to develop optimum assays for CL quantification by multiple reaction monitoring (MRM).

Using MRM quantification of PG and LPG levels, we tracked changes in the levels of these species during *in vitro* growth of *E*. *faecalis*. Previously, the levels of selected PG and PE species were monitored by MS in the Gram-positive *B*. *subtilis* and shown to remain constant during exponential growth. During stationary growth, the PG level decreased while the PE level increased. Neither the LPGs nor the glycolipids changed significantly during growth [82]. We show that the levels of different PGs change during logarithmic growth and entry into stationary phase. These PGs differ from each other both in terms of carbon chain length and the number of carbon-to-carbon double bonds, and thus our analysis reveals that *E*. *faecalis* favors PGs with specific chemical properties at different growth phases. Since higher LPG levels have been correlated with increased resistance to cationic antimicrobial peptides, the higher LPG levels observed in mid and late log phase *E*. *faecalis* may suggest that these growth phases may be most resistant to cationic antimicrobial peptides. Based on our DapR data, it is plausible that resistance to cationic antimicrobials may be further enhanced by a decrease in PG levels. *E*. faecalis might thus use a concomitant increase in LPG and decrease in PG as a strategy to resist CAMPs and daptomycin.

In this study, we have used a combination of mass spectrometry techniques to identify phospholipids and glycerolipids and we now have a detailed lipid profile for this organism. With the quantification of PG and LPG in hand, future studies are now possible to study lipid level dynamics under a variety of environmental conditions as well as exposure to antimicrobials. Thus, lipidomics has promising applications in the areas of microbiology, infectious diseases, and biofilms. We expect that lipidomics will play a pivotal role in the development of new and effective therapies against *E*. *faecalis* and other human pathogens.

## Supporting information

S1 Fig MS spectrum of (a) Diacylglycerols (DAGs) and (b) Triacylglycerols (TAGs) detected in *E*. *faecalis* strain OG1RF. Each peak is labelled with the class (DAG or TAG), fatty acid composition (e.g. 34:1), and the mass-to-charge (m/z) ratio (e.g. 612.5554 for DAG 34:1). The spectrum was acquired in positive ionization mode.
(TIF)

S1 Table Characterization of Phosphatidylglycerol (PG), lysyl-PG, Glycerophospho-Diglucosyl-Diacylglycerol (GPDGDAG), and Diglucosyl-Diacylglycerol (DGDAG) molecules of *E*. *faecalis* by mass spectrometry. (XLSX)

S2 Table Characterization of Cardiolipin (CL) molecules of *E*. *faecalis* by mass spectrometry. (XLSX)

S3 Table Characterization of Diacylglyerol (DAG) and Triacylglycerol (TAG) molecules of *E*. *faecalis* by mass spectrometry. (XLSX)

S4 Table Multiple Reaction Monitoring (MRM) transitions for Phosphatidylglycerol (PG) and lysyl-PG molecules of *E*. *faecalis*. (XLSX)
