# Supplementary figures and images for: Comprehensive analysis of phospholipids and glycolipids in the opportunistic pathogen Enterococcus faecalis
Source: PLoS One. 2017 Apr 19;12(4):e0175886. doi: 10.1371/journal.pone.0175886 (PMC5397010; doi:10.1371/journal.pone.0175886)

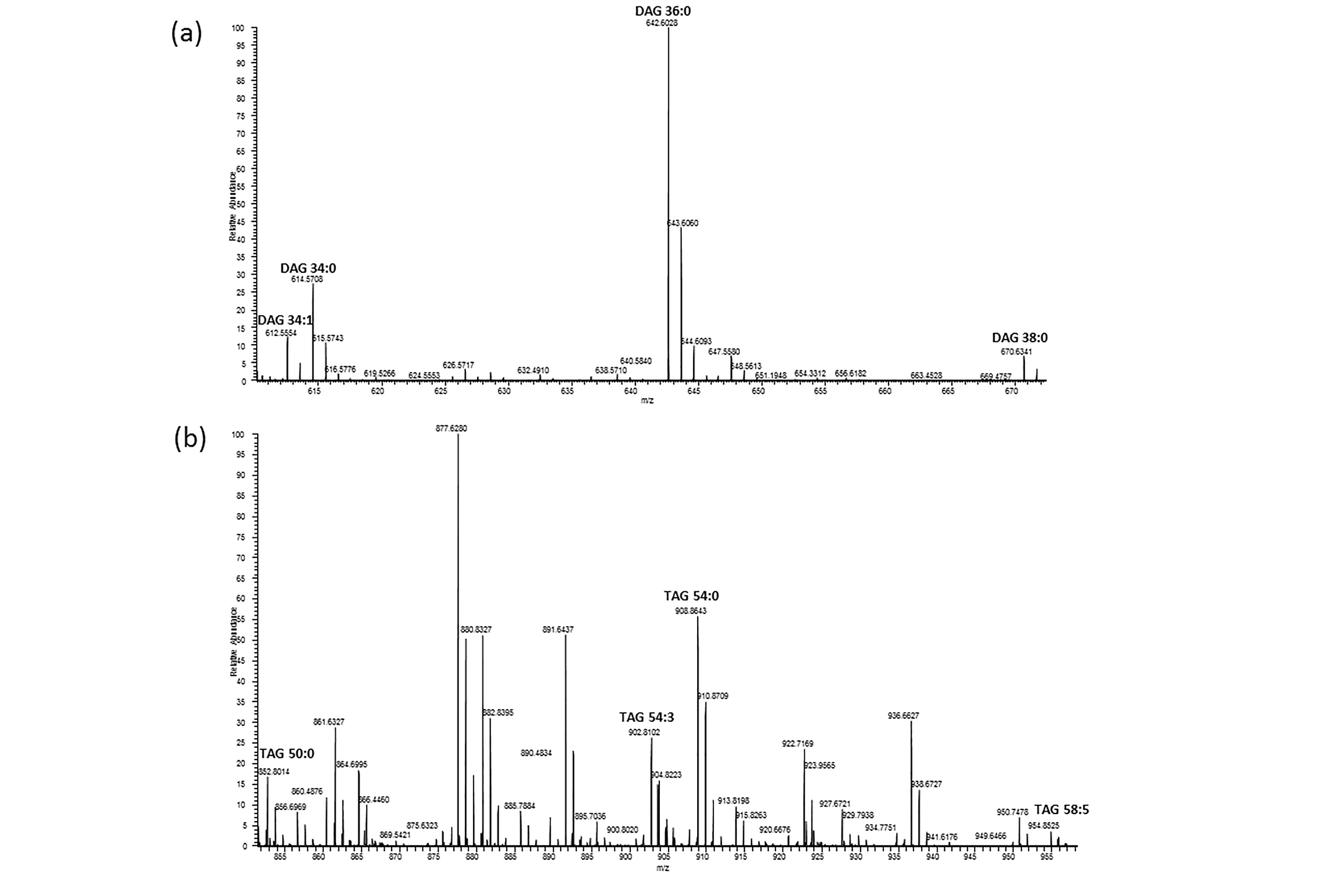

Supplement: S1 Fig — Each peak is labelled with the class (DAG or TAG), fatty acid composition (e.g. 34:1), and the mass-to-charge (m/z) ratio (e.g. 612.5554 for DAG 34:1). The spectrum was acquired in positive ionization mode. (TIF) [file pone.0175886.s001.tif]
